# Comparison of the behaviour of pro-oxidant additive containing plastic degradation in the unmanaged natural environment and in the laboratory

**DOI:** 10.1098/rsos.241270

**Published:** 2025-03-12

**Authors:** Fabiola Sciscione, Luba Prout, Jack W. E. Jeffries, Hajar J. Karam, Achilleas Constantinou, Fei Peng, S. M. Al-Salem, Helen C. Hailes, Mark Miodownik

**Affiliations:** ^1^ UCL Plastic Waste Innovation Hub, University College London, London, UK; ^2^ Department of Chemistry, University College London, London, UK; ^3^ Department of Biochemical Engineering, University College London, London, UK; ^4^ Environment & Life Sciences Research Centre, Kuwait Institute for Scientific Research (KISR), P.O. Box 24885, Safat 13109, Kuwait; ^5^ Department of Chemical Engineering, Cyprus University of Technology, Limassol 3036, Cyprus; ^6^ Mechanical Engineering Department, University College London, London, UK

**Keywords:** field trials, oxo-degradable, oxo-biodegradable, PAS 9017, pro-oxidants, microplastics

## Abstract

Pro-oxidant additive containing (PAC) plastics are designed to degrade in the unmanaged natural environment through oxidation and biological processes. In 2020, the British Standard Institution published the PAS 9017:2020 standard designed to ensure that PAC plastic tested under a specific set of protocols would successfully biodegrade in the environment. In this article, we compare the outcomes of laboratory tests carried out according to PAS 9017:2020 with field tests in an open unmanaged environment in the UK over 24 months. We report that the PAC cups were intact after 24 months and did not undergo significant abiotic degradation nor biodegradation during field tests. The PAC cups did undergo rapid abiotic degradation during accelerated UV laboratory tests, however the carbonyl index never reached 1.0. The molecular weight of the PAC cups decreased throughout the field trials and during the laboratory tests but neither satisfied the requirements stated in PAS 9017:2020. Earthworm avoidance tests and earthworm reproduction tests carried out in artificial soil showed no significant adverse effects or impact on the microbial community. We conclude that PAS 9017:2020 does not predict the real-world behaviour of the PAC plastics we tested in the open unmanaged environment in the temperate climate of the UK.

## 1. Introduction

Pro-oxidant additive containing (PAC) plastics is a term that describes a growing number of plastics which are designed to degrade in the unmanaged natural environment (open-air, soil and aquatic) through oxidation and other processes. These plastics are sold with various labels including ‘oxo-degradable’ plastics, ‘oxo-biodegradable’ plastics and ‘biodegradable’. It should be noted that not all plastics claiming to be biodegradable are PAC plastics, in fact only a minority are PAC plastics. Many other types of biodegradable plastics exist that employ different approaches to promoting biodegradation [1]. In an attempt to reduce this confusion, the term PAC has been coined to describe plastics that are intended to degrade via a particular set of mechanisms. Thus, PAC plastics are defined as polyolefin-based materials containing pro-oxidants such as transition metals (e.g. Fe, Co, Mn, Cu, Ce or Ni) in the form of salts (e.g. carboxylates, dithiodicarbamates and acetylacetonates) or organic complexes [1]. The additives act as catalysts in the polyolefin degradation processes of the polymer, reducing their chain length and causing the polymeric matrix to break down and the material to deteriorate. These processes are triggered by UV light (i.e. photo-induced degradation) combined with exposure to heat and oxygen. Eventually, the polymer degrades into smaller fragments of low molecular weight, which, it is claimed, can be assimilated by microorganisms [1]. This claim is disputed and has led to the banning of PAC plastics in some territories. For instance, in 2021, following an analysis of the available evidence about their impact on existing waste management systems and the rate of abiotic and biotic decomposition in the environment, the European Union banned the use of PAC plastics [2,3].

The testing standards to confirm the biodegradability of PAC plastics are ASTM D6954 [4] and BS 8472 [5]. In 2020, a new publicly available specification, PAS 9017 [6], was developed to provide methods, timescales and pass criteria to demonstrate that PAC plastics will biodegrade in the open-air terrestrial environment (i.e. littering or unmanaged disposal) without forming microplastics. PAS 9017 is divided into three parts:

- *weathering of polyolefins*: film or rigid polyolefin samples are subjected to laboratory accelerated weathering under specific conditions (light, air and heat) and timeframes specified in PAS 9017, which correspond to a specific time period of outdoor exposure under South Florida conditions. The rate of degradation is tested by carbonyl index (CI) and molecular weight analysis;
- *ecotoxicity testing*: on potential biologically hazardous substances present on the surface or in the polymer matrix; and
- *biodegradation of the wax after weathering*: in mesophilic conditions using soil as the medium for the test, as specified by BS EN ISO 17556 [7].

In 2023, a review of the literature showed that there is some evidence that abiotic degradation of PAC plastic occurs in hot dry climates but there is no evidence this occurs in cool or wet climatic regions such as the UK or under less ideal conditions (soil burial, surface soiling, etc.) [1]. They found there is little evidence of the successful biodegradability after abiotic degradation in any climate, hot, cold, wet or dry. Most PAC plastics studied in the literature showed biodegradability values in the range 5–60% and would not pass the criteria for biodegradability set by PAS 9017:2020. They highlighted the need for ecotoxicity studies to assess the possible effect of PAC additives and microplastic formation on the environment and biological organisms.

In this article, we report work we have undertaken to conduct ecotoxicity studies and biodegradability tests of a commercially available PAC plastic polypropylene (PP) beverage cup. We present work from our field studies of these PAC plastic cups in Dorset (UK) under conditions of the open unmanaged natural environment. We also report a set of laboratory tests on the same batch of PAC cups to test: (i) PAS 9017:2020 the abiotic degradation in a laboratory environment; (ii) results from laboratory-based biodegradability tests of these PAC cups; (iii) results of the same laboratory tests on PP cups of the same size and thickness as the PAC cups but made from virgin PP plastic with no additives; (iv) earthworm avoidance tests and earthworm reproduction tests carried out in artificial soil on PAC and virgin PP plastic; and (v) microbial analysis of the impact of biodegradation in soil under laboratory conditions.

## 2. Material and methods

### 2.1. Tested material

**Figure 1.**
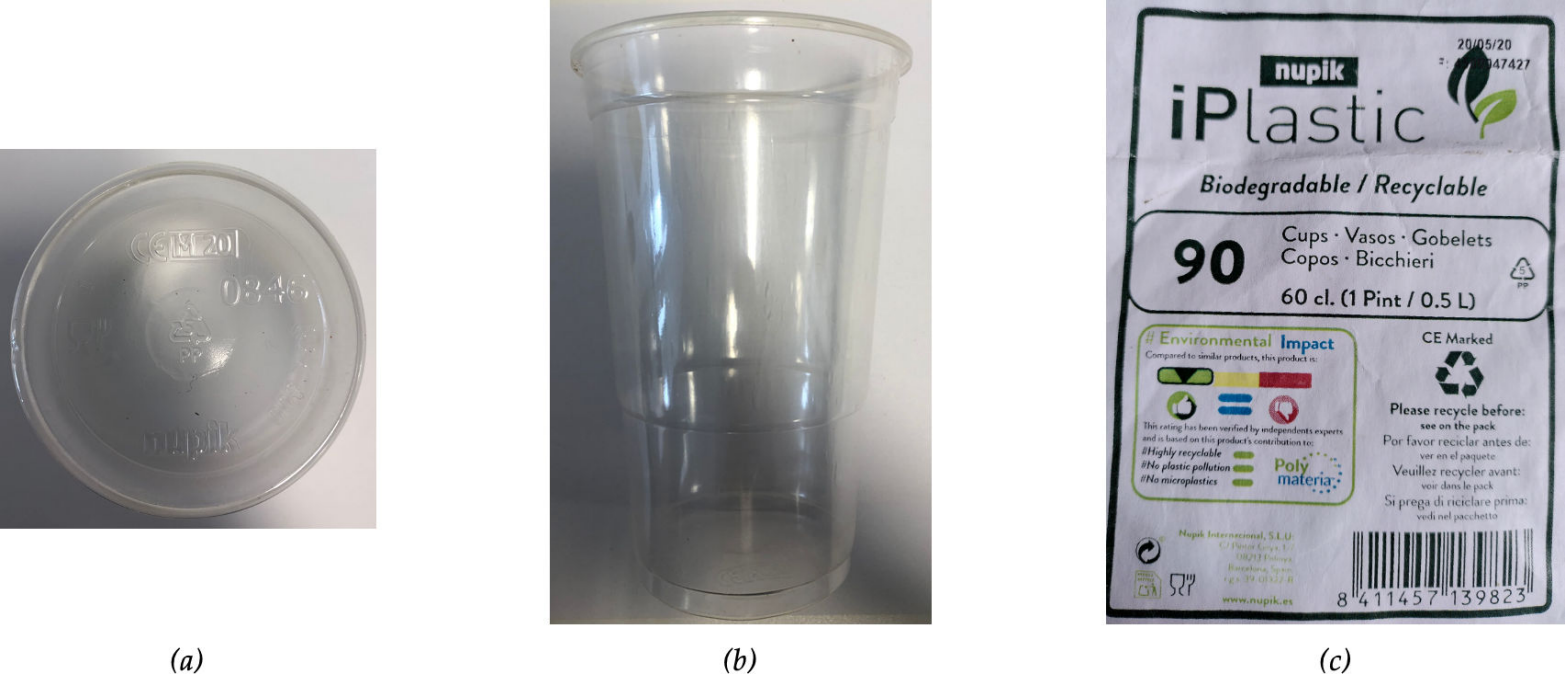
Commercially sourced PAC plastics on sale in the UK, purchased in December 2020. Transparent Nupik PP beverage cups doped with cycle+ additive (Polymateria Ltd, UK): (****a****) showing base of cup, (*b*) showing side-on, and (*c*) packaging of the set of cups.

The PAC plastics tested were Nupik PP plastic cups marketed under the tradename ‘i-Plastic’, which were purchased from a Costco outlet in December 2020. These cups were used as test materials for both field trial studies and laboratory tests (figure 1). According to the label details the cups were manufactured around May 2020 (figure 1). Table 1 shows the spectrographic analysis (inductively coupled plasma-optical emission spectroscopy (ICP-OES)) of two of the Nupik cups to determine the nature of the additives in the plastic, which shows the presence of transition elements Fe, Mn, Ni, Ti, and Zn. The significant disparity between the values may indicate poor mixing of the pro-oxidant additive during the manufacturing process. Virgin polypropylene (VPP) plastic drinking cups of the same size, shape and thickness were used as control materials for the UV-accelerated laboratory tests. The printed logo on the VPP plastic cups was avoided in all test specimens used in this work. The ‘unweathered’ Nupik PP and VPP plastic cups were stored in the dark at cool temperatures (4–10°C) and used as controls.

**Table 1.** Inductively coupled plasma optical emission spectroscopy (ICP-OES) results for two different PAC PP cups, labelled as ‘cup1’ and ‘cup2’ showing the elemental analysis of the cups indicating the presence of transition elements Fe, Mn, Ni, Ti, Zn. (The disparity between the values may indicate poor mixing of the match batch of cycle+ additive during the manufacturing process.)

detected elements	cup 1 (g 100 g^−1^)	cup 2 (g 100 g^−1^)
Ca	0.011	0.270
Fe	0.003	0.010
Mn	0.004	0.028
Ni	—	0.001
Ti	—	0.003
Zn	—	0.003

### 2.2. Outdoor exposure in real UK terrestrial environments

**Figure 2.**
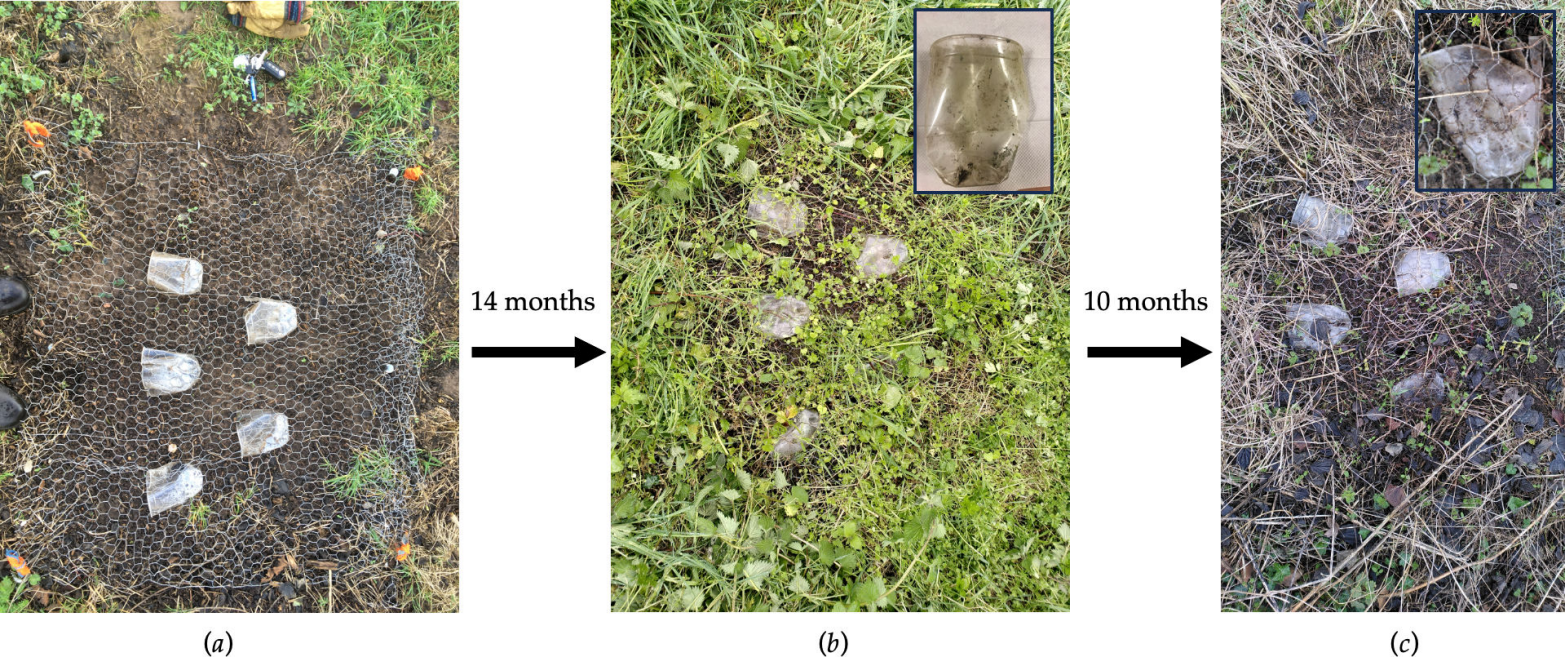
Photos showing field trials of five Nupik PP cups in a Dorset field. Five cups were positioned 15 cm apart on open ground and covered with chicken wire mesh to prevent being blown away in the wind. They were monitored for 24 months: (*a*) the initial set-up in Jan 2021; (*b*) showing the cups 14 months later and, May 2022, one of these (inset) was removed for laboratory testing; and (*c*) showing the remaining four cups 24 months later, and close up (inset).

Outdoor weathering tests were carried out on five Nupik PP plastic cups in an agricultural field in a rural location in Dorset, UK. The samples were placed on flat open ground cleared of vegetation in January 2021. They were placed 15 cm apart and covered with chicken wire mesh to prevent being blown away in the wind (figure 2*a*). The site was regularly inspected to make sure the cups were secure and that they were experiencing real-world conditions of sun, rain and the growth of grasses and other organisms around them. No pesticides or herbicides were used on the site.

### 2.3. UV-accelerated laboratory tests

**Figure 3.**
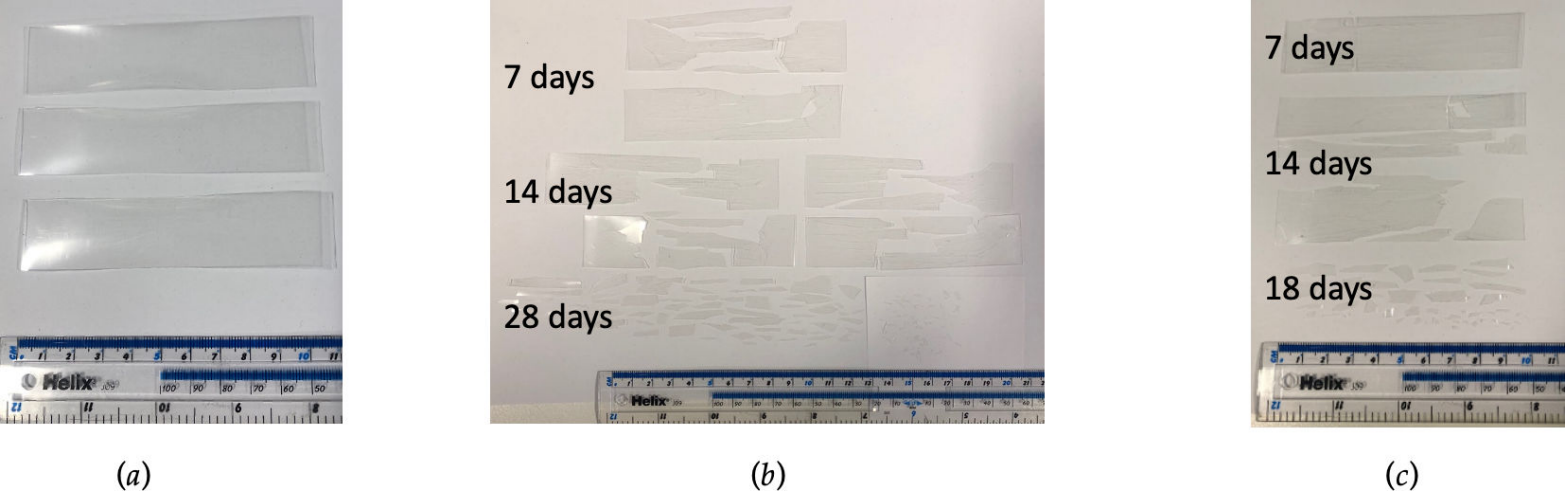
Accelerated laboratory tests carried out according to PAS 9017:2020 comparing the performance of Nupik PP cups with PP cups made from virgin plastic without additives. (*a*) shows the format and size of strips of cups cut out of VPP cups and PAC PP cups which were subject to accelerated laboratory testing. The Nupik PP and VPP samples were exposed to UV light for 28 days. Tests were carried out according to ASTM D4329 (cycle A) with 8 hours of continuous exposure at 60°C, followed by 4 hours of condensation at 50°C. At least three replicates were used in each exposure rack with an irradiance of 0.68 W m^−2^ and calibrated every 400 hours of continuous operation. (*b*) shows the physical appearance of the Nupik PP strips of cup after 7, 14 and 28 days exposure—the samples become embrittled and break up into microplastics. (*c*) shows the physical appearance of the virgin PP strips of cup after 7, 14 and 18 days exposure.

Nupik PP plastic cups and VPP plastic cups were cut into test specimens (10.5 cm × 2.5 cm) with a thickness of 0.213 ± 0.015 mm and 0.223 ± 0.011 mm, respectively (figure 3*a*). The test specimens were exposed to accelerated-laboratory controlled weathering using a Q-Lab QUV machine equipped with an UVA fluorescent lamp. Tests were carried out according to ASTM D4329 (cycle A) with 8 hours of continuous exposure at 60°C, followed by 4 hours of condensation at 50°C following past work reported in the literature [8,9]. At least three replicates were used in each exposure rack with an irradiance of 0.68 W m^−2^ and calibrated every 400 hours of continuous operation. Nupik PP and VPP samples were exposed to UV light until no further deterioration of the test samples was observed or until 28 days was reached, as stated in PAS 9017 for rigid samples. Both Nupik PP and VPP test specimens were retrieved from the weathering chamber at different time points during the UV-accelerated laboratory test for further analysis. Test specimens Nupik PP plastic cups were tested for 28 days with samples being removed for analysis at 7, 14 and 28 days (figure 3*b*). Test specimens from the VPP plastic cups were tested in an identical manner, but the last sample had broken up into microplastics by day 18 and was removed at this point (figure 3*c*).

### 2.4. Scanning electron microscopy

Scanning electron microscopy (SEM) was carried out with a Zeiss SEM Gemini 360. Samples were gold-coated with an Agar sputter coater set at 30 mA for 15 s before imaging. All images were processed using ImageJ software.

### 2.5. Carbonyl index measurements

Carbonyl index was assessed by attenuated total reflectance-Fourier transform spectroscopy (ATR-FTIR) following the PAS 9017:2020 guidelines. Measurements were carried out on a Shimadzu Infrared Tracer-100 between spectra range 4000 and 600 cm^−1^ with a resolution of 4 cm^−1^ and the number of scans set to 64. The CI was calculated according to PAS 9017:2020 by comparing the area under the band between 1850 and 1650 cm^−1^, corresponding to the carbonyl species formed during photo or thermo oxidation processes, to the area under the band between 1500 and 1420 cm^−1^, taken as a reference peak. An average of at least three measurements was taken on each side of the test specimen, named side 1 (outside of cup) and side 2 (inside of cup).

### 2.6. Molecular weight measurements

Molecular weight changes were analysed by high temperature-gel permeation chromatography (HT-GPC) on an Agilent PL GPC220 with a refractive index detector and a 3× Olexis PLgel column. The HT-GPC system was calibrated with Agilent Technology EasiVial PS-H polystyrene calibrants. Sample solutions were prepared based on BS ISO 16014. Samples were dissolved in 1,2,4-trichlorobenzene containing anti-oxidant at 190°C under agitation. The solutions were cooled down to 160°C, filtered through a 1.0 mm glass-fibre mesh and analysed at a flow rate of 1 ml min^−1^. All samples appeared to fully dissolve and to give clear solutions and no issues were encountered with filtration or chromatography of the solutions. Each sample was analysed by HT-GPC twice with both measurements showing high reproducibility.

### 2.7. Thermal analysis

Thermogravimetric analysis (TGA) was carried out using a Discovery TGA. Measurements were performed from 30°C to 600°C at a heating rate of 10°C min^−1^ under nitrogen atmosphere (flow rate of 25 ml min^−1^). Measurements were carried out in duplicate with 6−8 mg per sample. Weight loss (%) and differential thermogravimetric analysis (DTG, % °C^−1^) were recorded throughout the analysis, and thermogravimetric parameters such as the onset temperature (*T*_onset_) and maximum degradation temperature (*T*_max_) were extrapolated from the TGA and DTG curves, respectively.

Differential scanning calorimetry (DSC) curves were obtained with a Multi-Sample Discovery X3 DSC (TA Instrument) to gather information on the crystallinity of the analysed samples. DSC measurements were performed as a series of three consecutive scans under nitrogen atmosphere with 5−7 mg of sample and a heating/cooling rate of 10°C min^−1^. Samples were heated from 0°C to 230°C during the first scan. Subsequently, samples were cooled from 230°C to −20°C and finally heated from −20°C to 230°C. All measurements were repeated at least three times for each sample. Thermal parameters such as melting temperature (*T*_m_), melting enthalpy (Δ*H*_m_) and crystallinity (*χ*) were obtained from the DSC traces of the first and second heating scan. The crystalline content was determined by dividing the melting enthalpy by the melting enthalpy of a 100% crystalline PP (207 J g^−1^) [10].

### 2.8. Soil ecotoxicity tests

#### 2.8.1. Earthworm avoidance test

The earthworm avoidance tests were carried out by a commercial laboratory (Normec OWS nv, Pantserschipstraat 163, 9000 Gent, Belgium), which specializes in environmental testing and assessments.

The test method is based on ISO 11268-2 [11]. The purpose of the test is to evaluate the avoidance of earthworms from a test soil, to which a test item has been added in a specific concentration, towards an artificial soil to which no test item has been added. In the test, artificial soil (450 g) is placed in one half of a container, while test soil (450 g) is placed in the other half. A plate is initially used to separate both compartments. At the start of the test, the plate is removed, and 10 earthworms are placed on the soil surface at the boundary of the two compartments. Fresh cow dung (5 g) is placed on the soil surface in the centre of both compartments. After an incubation period of 48 hours, the number and weight of the earthworms on each side of the test container are recorded. Also, the mass of the remaining cow dung is determined.

The VPP and Nupik PP cups were crushed into a powder using a pestle and mortar. In each tray, artificial soil was placed in one half of a container, while test soil with 1 g kg^−1^ (on a dry weight basis) of the test item was placed in the other half. A plate was initially used to separate both compartments. At the start of the test, the plate was removed, and 10 earthworms were placed on the soil surface at the boundary of the two compartments. Fresh cow dung was placed on the soil surface in the centre of both compartments. At the end of the test (48 hours), the number of earthworms was determined for each compartment separately.

The dry matter was determined by drying at 105°C for at least 14 hours and weighing, as described in ISO 11268-2 ‘M_009. Determination of moisture content’. The dry matter was determined as a percentage of the wet weight. The pH was measured with a pH meter after calibration with standard buffer solutions (pH = 4.00, pH = 7.00 and pH = 10.00), as described in ISO 11268-2 ‘M_006. Determination of pH and electrical conductivity’ [11]. Before inserting the electrode, the sample was diluted with distilled water at a ratio of 5 : 1 (five parts demineralized water to one part sample) and thoroughly mixed, as described in ISO 11268-2 ‘M_057. Extraction of water and potassium chloride soluble nutrients and elements’ [11]. During the test, two types of balances were used: a Sartorius ACCULAB ATILON with internal calibration (max. 220 g, *d* = 0.1 mg) for determining the weight of the earthworms and the test item, and a Sartorius AX6202 (max. 6200 g, *d* = 0.01 g) for weighing the artificial soil.

#### 2.8.2. Earthworm reproduction test

The earthworm reproduction tests were carried out by a commercial laboratory (Normec OWS nv, Pantserschipstraat 163, 9000 Gent, Belgium), which specializes in environmental testing and assessments.

The test method is based on ISO 11268-2 [11]. The VPP and Nupik PP cups were crushed into a powder using a pestle and mortar. These powders were tested in a concentration of 1 g kg^−1^ (on a dry weight basis). The test item was added to artificial soil to obtain the desired test concentration. The obtained mixture was thoroughly mixed before being used for the earthworm test. The earthworm reproduction test was carried out in 1000 ml glass jars, each containing 500 g (dry mass) of a mixture of artificial soil and test item. Each series was tested in at least three replicates. Additionally, the pure artificial soil was tested in at least three replicates. The details of the 56 days experimental procedure are itemized in the electronic supplementary material, table S1. After 28 days, the survival and the live weight of the earthworms in each reactor were determined. After 56 days, the pH and the number of offspring per test container hatched from the cocoons were determined. The toxicity of possible residuals of the test item was evaluated by comparing the survival, mean weight and reproduction of earthworms in the artificial soil/test item mixture to those in the pure artificial soil. Survival and mean weight of the worms were assessed 28 days after application. The effect on reproduction, as the definitive end point, was measured by counting the number of offspring hatched from the cocoons after an additional period of four weeks (56 days after application).

The dry matter was determined by drying at 105°C for at least 14 hours and weighing, as described in ISO 11268-2 ‘M_009. Determination of moisture content’ [11]. The dry matter was given as a percentage of the wet weight. The pH was measured with a pH meter after calibration with standard buffer solutions (pH = 4.00, pH = 7.00, and pH = 10.00), as described in ISO 11268-2 ‘M_006. Determination of pH and electrical conductivity’ [11]. Before inserting the electrode, the sample was diluted with distilled water at a ratio of 5 : 1 (five parts demineralized water to one part sample) and thoroughly mixed, as described in ISO 11268-2 ‘M_057 Extraction of water and potassium chloride soluble nutrients and elements’ [11].

The water holding capacity (WHC) was determined by measuring the mass of water evaporating from the soil saturated with water when dried to constant mass at 105°C. This amount divided by the dry mass of the soil gives the total WHC. This analysis was performed in triplicate, as described in ISO 11268-2 ‘M_053. Determination of the water holding capacity of a soil’ [11]. During the test, two types of balances were used: a Sartorius ACCULAB ATILON with internal calibration (max. 220 g, *d* = 0.1 mg) for determining the weight of the earthworms and the test item, and a Sartorius AX6202 (max. 6200 g, d = 0.01 g) for weighing the artificial soil.

### 2.9. Microbial and metagenomic DNA analysis

**Figure 4.**
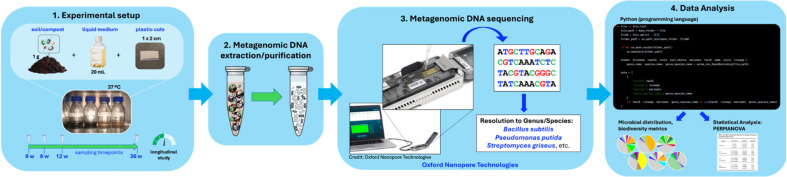
A schematic illustrating the experimental process for analysing microbial distribution in the samples.

The aim of this analysis was to assess the effect of biodegradation, by microorganisms found in soil samples, on the microbial community composition among the three treatment groups (control soils, soils with Nupik PP, and soils with VPP). An overview of the analysis method is shown in figure 4.

#### 2.9.1. Reagents

All reagents were obtained from Merck Millipore or Thermo Fisher Scientific, unless stated otherwise.

#### 2.9.2. Soil and plastic samples

Industrial compost 1 (IC1), industrial leachate 1 (IL1), industrial compost 2 (IC2), industrial leachate 2 (IL2), home compost 1 (HC1), home compost 2 (HC2), Dorset soil 1 (DS1) and Dorset soil 2 (DS2) were sourced from various industrial and residential locations, including composting facilities and soil sites in Dorset at the field test site for the PAC plastic cups.

#### 2.9.3. Soil enrichment setup

Individual soil/compost or leachate samples (1 g or 1 ml) were added to sterilized 250 ml Duran bottles containing 20 ml of one-quarter strength nutrient broth (Oxoid™, No. 2). Nupik PP and VPP cups, cut into 1 cm × 2 cm pieces (referred to as PACs and VPP samples), were sterilized in 100% ethanol for an hour, then washed in 70% ethanol and allowed to dry under sterile air. These samples were placed into the bottles using sterile tweezers and statically incubated at 37°C or 50°C for up to 36 weeks to investigate their effect on soil microorganisms. Control samples, identically prepared but excluding the plastic samples, were run concurrently. In this set-up, each soil/compost or leachate sample was incubated with a Nupik PP sample, VPP sample and without plastic, for comparative analysis. A 1 ml sample of the soil-containing solution was taken at 8, 12 and 36 weeks and stored at −80°C until needed.

#### 2.9.4. Metagenomic DNA purification

Metagenomic DNA (mDNA) was extracted and purified from 1 ml soil-containing samples using a Mag-Bind^®^ Environmental DNA 96 Kit (Omega Bio-tek, Inc., Norcross, GA, USA). The samples were added to 15 ml centrifuge tubes pre-filled with ceramic beads from one well of an E-Z 96 Disruptor Plate and 1050 μl of SLX-Mlus buffer. The samples were vortexed at maximum speed for 5 min, then supplemented with 2 μl of RNase A and 20 μl of lysozyme (at 50 mg ml^−1^) and incubated at 37°C for 20−30 min. Following incubation, 350 μl of the DS buffer was added and the samples were vortexed again for 5 min. Additionally, 100 μl of resuspended activated charcoal in pure water was added to the samples at a final concentration of 0.5% v/v. The samples were vortexed briefly and then incubated at 70°C for 10 min and subsequently at 95°C for 2 min. Afterwards, the samples were centrifuged at 2500*g* for 10 min at room temperature. The supernatant from each sample was transferred into two new 2 ml microcentrifuge tubes, distributing approximately 1 ml into each. Prechilled P2 buffer (134 μl) and cHTR reagent (200 μl) were added and the samples vortexed and centrifuged at 10 000*g* for 5 min at room temperature. Up to 800 μl of the supernatant was transferred to fresh microtubes, resulting in three tubes/fractions per sample. An equal volume of XP1 buffer and 20 μl of Mag-Bind^®^ Particles RQ were added to each. The samples were shaken in a ThermoMixer at 2000 rpm for 5 min at room temperature. The tubes were then placed on a magnetic separation device until all particles migrated to one side of the tube wall. The cleared supernatant was removed, and then the second (and third) fraction of the same sample was combined. After being removed from the magnetic device, the samples were washed with 500 μl of VHB buffer and vortexed for 1 min at room temperature. The tubes were placed on the magnetic separation device again, and the supernatant was removed. This wash step was repeated twice more using 800 μl of 70% ethanol. After the final wash, the tubes were left on the magnetic device for an additional minute, after which any residual liquid was removed. The tubes were placed in a ThermoMixer at 50°C for 15 min without shaking to dry the samples. Preheated elution buffer (55 μl) at 70°C was then added to elute the mDNA. The tubes were incubated in the ThermoMixer at 50°C with shaking at 2000 rpm for 5 min, and then placed on a magnetic separation device until all particles migrated to one side of the tube wall. The cleared solution was transferred to a new microtube, using 1 μl for analysis with the NanoDrop^®^ 2000c (Thermo Scientific) and another 1 μl for analysis with the Qubit 4 Fluorometer (Invitrogen) to determine DNA concentration and purity. The samples were then stored at −20°C until needed.

#### 2.9.5. Metagenomic DNA sequencing

mDNA sequencing was performed using the Oxford Nanopore Technologies (ONT; Oxford, UK) portable sequencer, MinION, using their Rapid Barcoding Kit 96 V14 (SQK-RBK114.96). The ONT MinKNOW software (23.11.4 release) enabled the conversion of the raw data into basecalled reads, which were subsequently demultiplexed using the ONT EPI2MEAgent.

#### 2.9.6. Sequencing results analysis

Sequencing data, formatted in comma separated values (CSV), were parsed and analysed using Python (v. 3.11). To provide insights into temporal changes in bacterial communities, a diversity analysis was performed. Alpha diversity metrics were used to assess the richness of genera within a functional community at various time points. Additionally, Shannon diversity, which considers both richness and evenness, was used to examine the taxonomic composition, or proportional abundances of genera, across different time points. To identify patterns in the distribution of bacterial genera, the composition of microbial communities, specifically focusing on genera that constituted ≥1% of the total in a sample, was visualized using pie charts. To investigate the impact of treatment on microbial community composition, PERMANOVA, a statistical analysis method, was employed. This approach assessed whether there were differences in the microbial community composition among the three treatment groups—control soils, soils with PACs and soils with VPP—at individual time points. The method was also used to explore the influence of time on microbial community composition.

## 3. Results

### 3.1. Visual and mechanical inspection

#### 3.1.1. Outdoor weathering tests

A Nupik PP cup was retrieved from the Dorset field site after 14 months of outdoor exposure. The cup was discoloured but was mechanically intact (figure 2*b*). At 24 months, the four remaining cups were inspected and shown to still be discoloured and cracked, but mechanically intact (figure 2*c*).

#### 3.1.2. UV-accelerated laboratory tests

The level of abiotic degradation observed for both UV-aged Nupik PP and VPP test specimens was compared to that of their respective ‘unweathered’ controls (figure 3). Nupik PP test specimens visually showed severe cracking and breakdown into smaller fragments after 7 days of UV exposure (figure 3*b*). After 14 and 28 days the test specimens showed signs of further mechanical fragmentation, forming large microplastics (1−5 mm) after 28 days of UV exposure. VPP specimens exposed to UV-accelerated ageing for 7 days were visibly intact and did not show any signs of degradation (figure 3*c*). After 14 days of continuous UV exposure, VPP samples started showing some cracking and breakdown into a few smaller fragments with dimensions around 1 cm. Severe degradation was observed after 14 days of continuous UV exposure. By day 18, the samples had fully disintegrated into large microplastics.

### 3.2. Scanning electron microscopy analysis

SEM analysis was carried out on the Nupik PP and VPP samples after UV-accelerated ageing. These were compared with SEM analysis of samples from outdoor exposure and unweathered samples.

**Figure 5.**
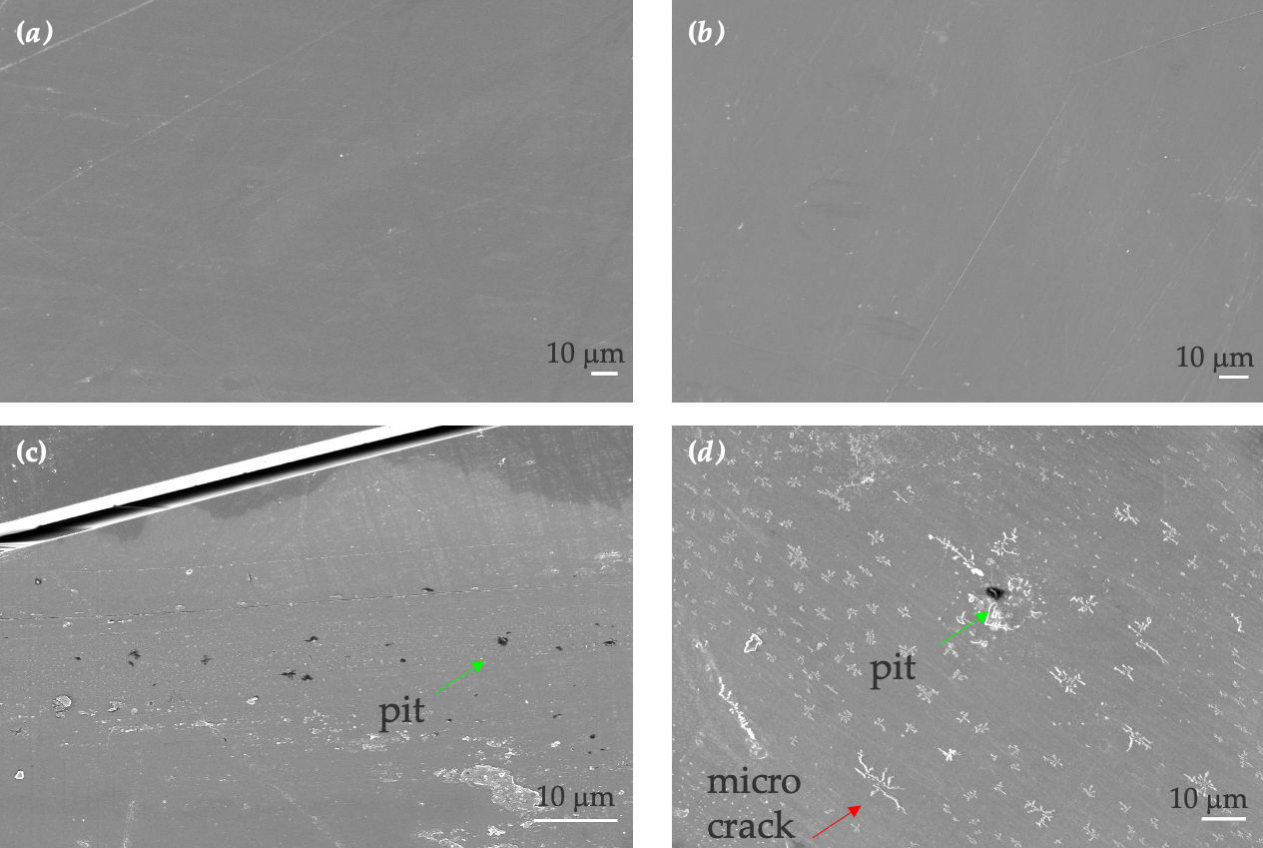
SEM images of Nupik cup plastic samples. (*a*) Unweathered control. (*b*) Field trial. (*c*) Artificially aged samples after 7 days of UV exposure. (*d*) ArtificIally aged sample after 28 days of UV exposure. Scale bar is 10 μm in all images. Green and red arrows highlight the presence of pits and microcracks respectively.

After 14 months of outdoor exposure, the Nupik PP field trial sample did not show any signs of degradation and the morphology of the surface could not be distinguished from that of the unweathered control sample (figure 5*a*,*b*). After 7 and 28 days of UV exposure, formations of pits and propagation of microcracks were observed on the surface of the sample (figure 5*c*,*d*). This could be associated with stress release induced by chain scission as a result of the photo-oxidation process [12].

**Figure 6.**
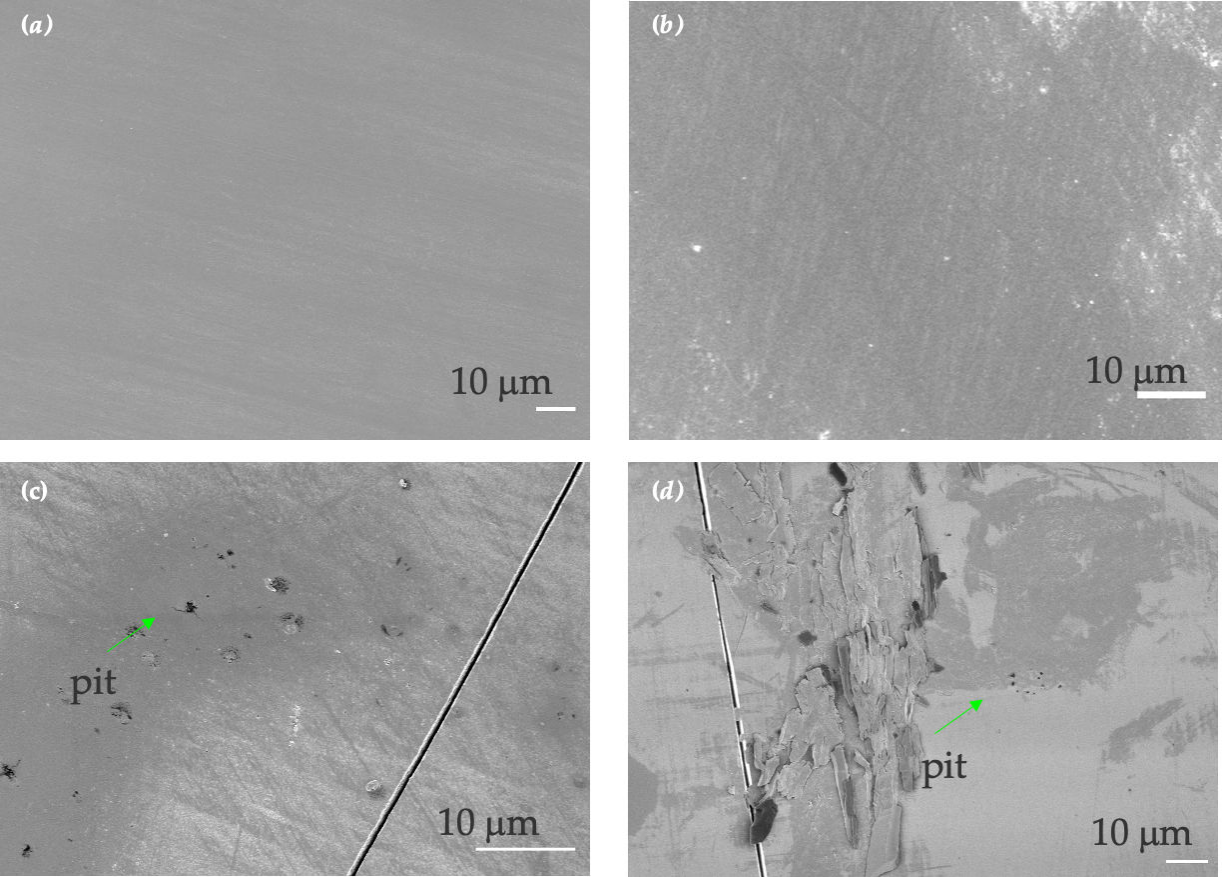
SEM images of VPP plastic samples. (*a*) Unweathered control. (*b*) Artificially aged samples after 7 days of UV exposure. (*c*) Artificially aged sample after 14 days of UV exposure. (*d*) Artificially aged sample after 18 days of UV exposure. Scale bar is 10 μm in all images. Green arrows highlight the presence of pits.

After 7 days of UV exposure, VPP samples showed some surface erosion compared to the unweathered VPP control, but no degradation was detected (figure 6*a*,*b*). After 18 days of UV exposure, degradation of the surface increased with the formation of pits, and deformation of the surface could also be observed. No microcracks were observed for the UV-aged VPP (figure 6*c*,*d*).

### 3.3. Carbonyl index

**Figure 7.**
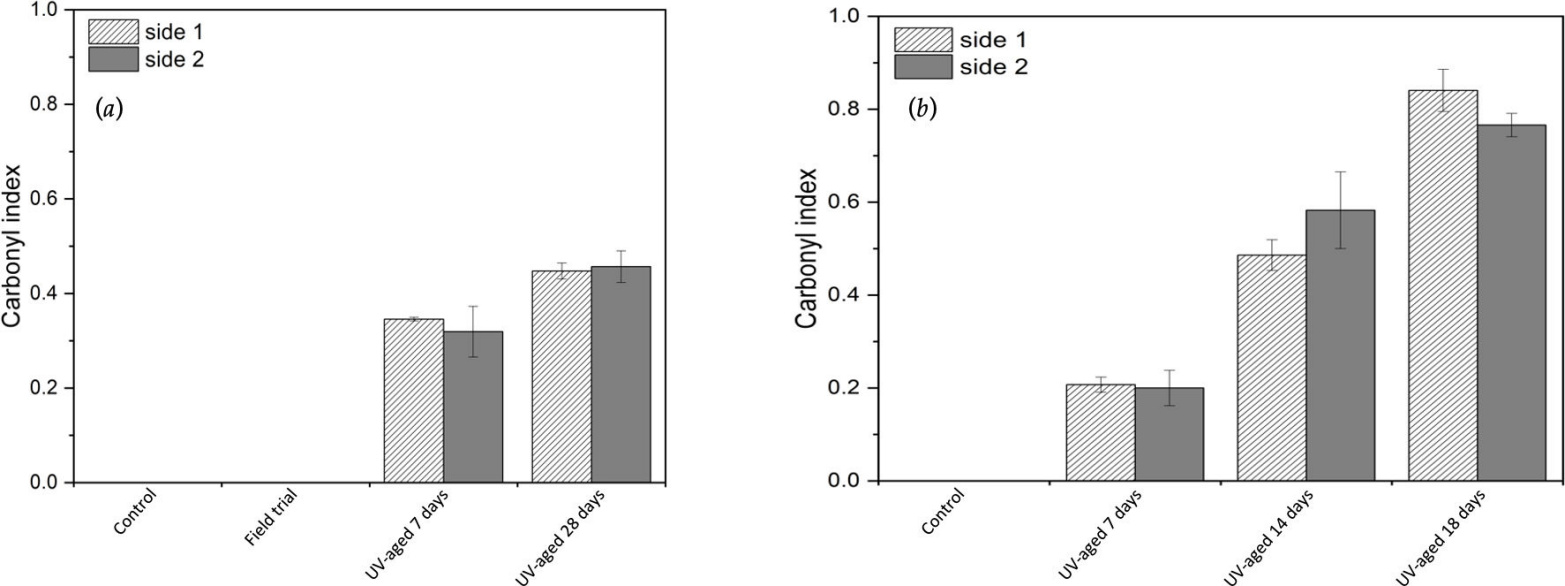
Carbonyl index (CI) assessed by attenuated total reflectance-Fourier transform spectroscopy (ATR-FTIR) following the PAS 9017 guidelines: (*a*) Nupik PP plastic samples; and (*b*) VPP plastic samples. According to PAS 9017, the CI value should be higher than 1.0 for a polymer to be qualified for soil biodegradability. Neither sample reaches this.

CI measurement was used to assess the extent of oxidation of polyolefins following artificial or outdoor weathering. The CI values were determined from the ATR-FTIR spectra for the UV-accelerated and outdoor-weathered samples considered in this study (electronic supplementary material, figures S1 and S2) and are reported in figure 7 with their respective controls. The unweathered Nupik PP control samples measured a CI value of zero (figure 7). The same behaviour was observed for the 14 months weathered field trial sample. Figure 7 also shows that an increase of the CI value was observed only upon accelerated UV testing for both the Nupik and VPP samples. Surprisingly, the VPP samples showed higher values of CI within shorter UV exposure times (14 and 18 days) compared to the UV-aged Nupik PP samples (28 days). However, neither the Nupik PP nor the VPP samples reached CI values above 1.0, which, according to PAS 9017, is the CI value required for a polymer to be qualified for soil biodegradability.

### 3.4. Molecular weight analysis

Changes in molecular weight were used to assess whether the oxidized and degraded polymers can be classified as soil biodegradable. PAS 9017 criteria are: *M*_n_ < 5000 Da; *M*_w_ loss > 90%; *M*_z_ < 30000 Da. The *M*_w_, *M*_w_ loss%, *M*_n_, *M*_z_ and PdI values measured for all the analysed samples are reported in table 2.

**Table 2.** Molecular weight analysis by high temperature gel permeation chromatography of VPP and Nupik PP. (Based on these results neither the PAC PP nor the VPP samples satisfy the requirements in terms of *M*_w_, *M*_n_, *M*_z_ stated in the PAS 9017 after either UV-accelerated laboratory tests or 14 months outdoor exposure.)

sample	run	*M*_w_ (Da)	*M*_w_ loss (%)	*M*_n_ (Da)	*M*_z_ (Da)	PdI
Nupik PP control	1 2	297 211 295 446		56 668 51 106	828 164 852 328	5.2 5.8
Nupik PP UV-aged 7 days	1 2	30 633 31 232	89.7 89.5	11 696 11 790	56 658 59 826	2.6 2.6
Nupik PP UV-aged 28 days	1 2	44 752 46 011	84.9 84.5	12 881 13 451	156 293 156 551	3.5 3.4
Nupik PP field trial	1 2	54 252 53 938	81.7 81.8	17 911 16 480	110 338 111 563	3.0 3.3
VPP control	1 2	337 756 356 301		85 220 78 115	973 973 1 120 000	4.0 4.6
VPP UV-aged 7 days	1 2	253 916 253 652	26.8 26.9	56 532 54 554	658 930 652 763	4.5 4.6
VPP UV-aged 14 days	1 2	77 743 71 118	77.6 79.5	13 432 12 632	477 742 407 793	5.8 5.6
VPP UV-aged 18 days	1 2	33 621 33 750	90.3 90.2	11 375 11 654	69 696 69 961	3.0 2.9

The VPP cup samples showed a continuous decrease in *M*_n_ and *M*_z_ (increase in *M*_w_ loss%) during accelerated ageing tests. The *M*_w_ loss was 26.9% by day 7 of accelerated ageing, 78.6% by day 14 of accelerated ageing and 90.3% by day 18. The molecular weight distribution curves show that a normal distribution of molecular weight was maintained as the sample shifted to smaller *M*_w_ (electronic supplementary material, figure S3).

The Nupik PP cup samples behaved differently. They showed a dramatic initial decrease in *M*_n_ and *M*_z_ (increase in *M*_w_ loss%). The *M*_w_ loss was 89.6% by day 7 of accelerated ageing. However, after 28 days of accelerated ageing, there was an increase in *M*_w_, *M*_n_, and *M*_z_. By the end of the test, the overall loss of molecular weight was 84.7%. From the molecular weight distribution curves of the 28 day UV-aged Nupik PP cup samples (electronic supplementary material, figure S3), it appears evident that this increase in the average reflects a broadening of the distribution of *M*_w_, hinting perhaps that cross-linking might be occurring in the sample. Table 2 also shows the results of the field-tested (14 months) Nupik PP cup sample. The sample reached a *M*_w_ loss of 81.8% by 14 months. The molecular weight distribution is similar to the UV-aged Nupik PP cup samples (electronic supplementary material, figure S3).

Based on the above results, the Nupik PP does not satisfy the requirements in terms of *M*_w_, *M*_n_ and *M*_z_ stated in PAS 9017 after either UV-accelerated laboratory tests or 14 months outdoor exposure.

### 3.5. Thermal analysis

#### 3.5.1. Thermogravimetric analysis

The thermal properties of Nupik PP and VPP cup samples were studied by TGA, from which parameters such as the onset temperature and maximum decomposition temperature can be obtained to understand the thermal behaviour of polymers. The onset temperature (*T*_onset_) is defined as the temperature at which weight loss and thermal decomposition of the polymer begin. The maximum decomposition temperature (*T*_max_) is associated with the temperature at which thermal degradation occurs at the maximum degradation rate.

The shape of the TGA curves for the Nupik PP samples did not change after UV or outdoor weathering and displayed a one-step degradation behaviour (electronic supplementary material, figure S4). A decrease of *T*_onset_ was observed only for the UV-aged samples. No significant differences were observed in the values of *T*_max_ of the UV-aged, field trial, and control samples (electronic supplementary material, table S2).

A more evident thermal destabilization was observed for the UV-aged VPP samples, with *T*_onset_ and *T*_max_shifting towards lower values with increased exposure times (electronic supplementary material, figure S4 and table S2). The different thermal behaviour could be attributed to the presence of a higher fraction of lower molecular weight oxidation products in the VPP samples compared to the Nupik PP samples, as already seen in their molecular weight distribution curves. This low molecular weight fraction is a result of the chain scission reactions that take place during photo-oxidation and shows lower decomposition temperatures [13].

#### 3.5.2. Differential scanning calorimetry

**Figure 8.**
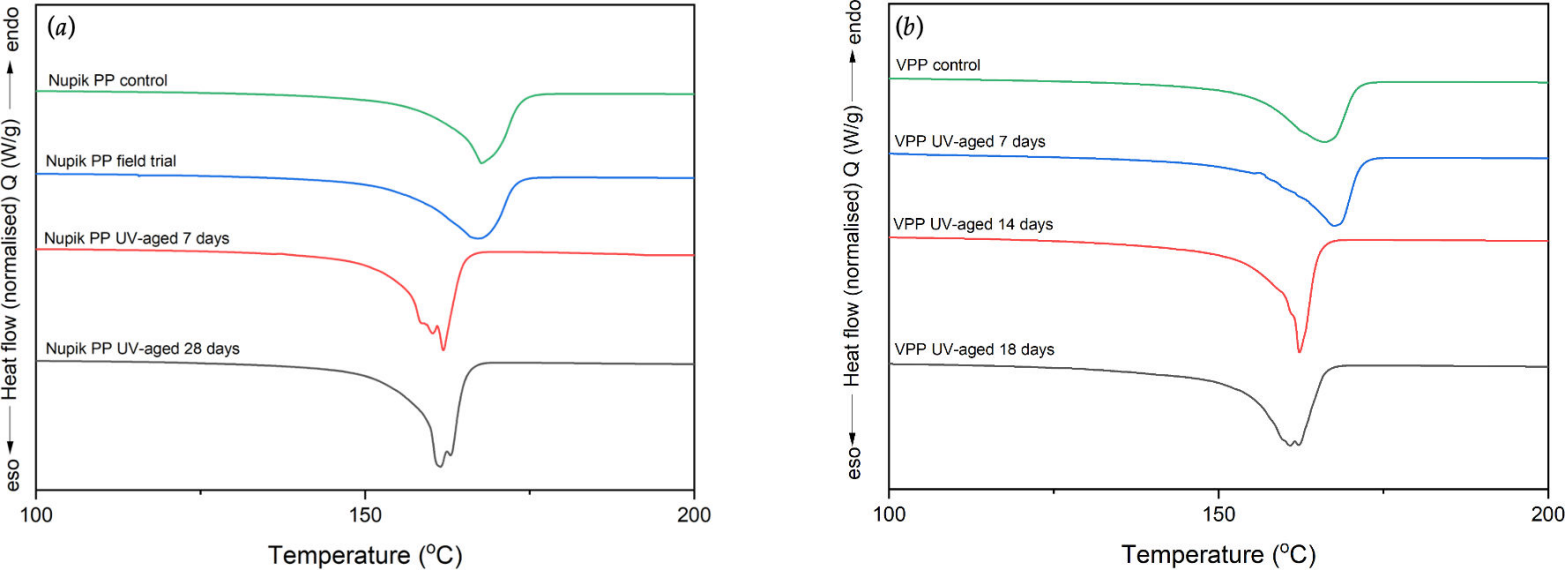
DSC curves: (*a*) Nupik PP plastic cup samples; and (*b*) VPP plastic cup samples. The endotherm peaks of Nupik PP control and Nupik PP field trial samples are similar indicating a similar degree of crystallinity, while the artificially aged samples behaviour differently in both VPP and PAC PP samples, exhibiting a bifurcation of peaks.

DSC was performed to measure the melting behaviour of the Nupik PP and VPP samples after accelerated UV and outdoor weathering, and the results were compared to their respective controls (figure 8). The two types of materials showed broadly similar behaviour with one single endothermic peak and a *T*_onset_ slightly shifting towards lower temperatures as the duration of artificial ageing increased. One difference did emerge at higher ageing times, where the DSC traces showed a multimodal behaviour with multiple melting peaks and *T*_max_ values below 160°C. These peaks occurred at day 7 for the Nupik PP samples and at day 18 for the VPP samples. Overall, laboratory UV exposure and higher temperatures seemed to induce faster degradation compared to 14 months of outdoor weathering (table 3).

**Table 3.** Differential scanning calorimetry data for Nupik PP and VPP samples.

first heating ramp				
sample	*T*_onset_ (°C)	*T*_m_ (1st peak; °C)		*χ* (%)
Nupik PP control	159.1 ± 3.2	167.8 ± 0.2		49.5 ± 2.5
Nupik PP field trial	158.7 ± 3.1	167.5 ± 0.4		48.1 ± 2.9
Nupik UV-aged 7 days	159.0 ± 1.6	161.7 ± 0.7		48.1 ± 4.6
Nupik UV-aged 28 days	158.7 ± 1.9	162.6 ± 1.7		49.8 ± 3.3
VPP control	155.2 ± 1.3	166.0 ± 0.7		51.5 ± 1.0
VPP UV-aged 7 days	156.1 ± 1.8	167.0 ± 0.5		51.0 ± 3.2
VPP UV-aged 14 days	159.1 ± 1.8	162.7 ± 1.3		52.9 ± 3.1
VPP UV-aged 18 days	158.4 ± 4.2	155−160^a^		51.5 ± 4.7

second heating ramp				
sample	*T*_onset_ (°C)	*T*_m_ (1st peak; °C)	*T*_m_ (2nd peak; °C)	*χ* (%)
Nupik PP control	155.0 ± 0.3	164.4 ± 0.5		48.8 ± 1.7
Nupik PP field trial	152.9 ± 1.4	163.3 ± 0.4		50.2 ± 0.8
Nupik UV-aged 7 days	144.2 ± 0.4	157.2 ± 0.1	148.7 ± 0.1	48.2 ± 0.6
Nupik UV-aged 28 days	144.4 ± 1.6	158.3 ± 1.7	149.8 ± 1.9	47.9 ± 1.4
VPP control	153.3 ± 0.2	163.4 ± 0.3		49.6 ± 0.6
VPP UV-aged 7 days	153.3 ± 0.3	162.9 ± 1.0		50.2 ± 0.7
VPP UV-aged 14 days	144.5 ± 7.3	158.5 ± 3.8	150.7 ± 0.8	50.1 ± 0.8
VPP UV-aged 18 days	141.9 ± 2.6	156.8 ± 1.0	148.9 ± 0.6	47.4 ± 1.0

### 3.6. Biodegradability

Neither the PAC nor VPP plastics biodegraded in our laboratory tests within the observed period (approximately 8.3 months), nor did they produce a statistically significant impact on the soil microbiome over this timeframe (electronic supplementary material, figures S5–S14). Statistical analysis revealed no significant differences in microbial community composition among the three treatment groups (control soils, soils with PACs and soils with VPP) at individual time points (i.e. 8, 12 and 36 weeks) (electronic supplementary material, tables S3 and S4). However, given the observed changes in values over time, there is potential for greater differentiation between the treatment groups over an extended incubation period.

### 3.7. Ecotoxicity tests

#### 3.7.1. Earthworm avoidance test

Powdered VPP and Nupik PP cups were tested for their impact on earthworm avoidance behaviour at a concentration of 1 g kg^−1^ (on a dry weight basis). In both test soils, no significant avoidance behaviour was observed in the test soil compartment. A similar or even slightly higher number of earthworms was present in the test soil compartment when compared to the artificial soil compartment (table 4). Based on these results, it can be concluded that no significant avoidance of earthworms from the test soil compartment to the artificial soil compartment was observed.

**Table 4.** Earthworm avoidance test as part of toxicity testing of VPP and Nupik PP cups. (Powdered cups at a concentration of 1 g kg^−1^ on dry weight basis added to soil (three test concentrations in three replicates). )

additive	presence of earthworms per compartment (%)	
	soil without additive	soil with additive
none	40 ± 20	60 ± 20
VPP	50 ± 0*	47 ± 6^a^
Nupik PP	37 ± 12	63 ± 12

^a^ The sum of both compartments is <100% because one dead earthworm was retrieved on the surface of compartment B (= test soil compartment). No significant avoidance of soil with additives by earthworms was observed.

#### 3.7.2. Earthworm reproduction test

An earthworm reproduction test was performed on powdered VPP and Nupik PP cups at a concentration of 1 g kg^−1^ (on a dry weight basis). The validation criteria according to ISO 11268-2 [11] comprise three elements: (i) survival of worms in the artificial soil series should be >90% (we measured 100% survival); (ii) the rate of production of juveniles should be >30 per control container (we measured an average rate of production of juveniles of 59); and (iii) the coefficient of variation of the reproduction in the control series should not exceed 30% (we found the coefficient of variation of reproduction in the artificial soil to be 14%). As a consequence, we concluded that our test was valid.

**Figure 9.**
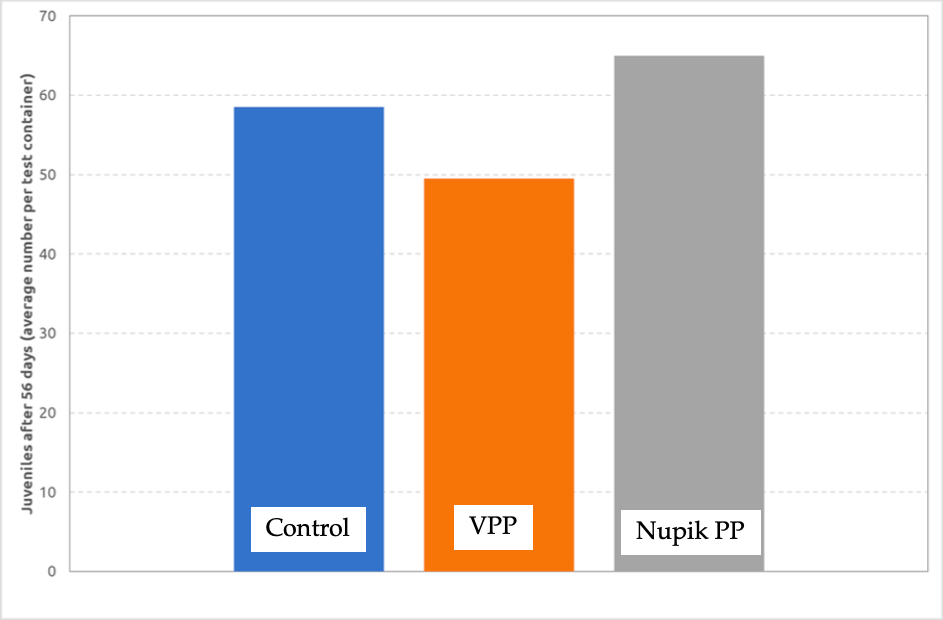
Earthworm reproduction test as part of toxicity testing of VPP and Nupik PP cups. Powdered cups at a concentration of 1 g kg^−1^ on dry weight basis added to soil, (three test concentrations in three replicates). No statistically significant negative effect on the survival and the mean weight of adult earthworms was observed in either case.

Complete survival was observed in the test series in both cases (figure 9). Moreover, the weight of the earthworms increased in both test series during the test. The live weight yield (as % of start) in both test series was comparable to or higher than the live weight yield (as % of start) in the artificial soil series. No significant negative effect on the survival and mean weight of adult earthworms was observed in either case.

A lower number of juveniles was present in the test series with powdered VPP compared to the artificial soil series (relative value: 85%), while a higher number of juveniles was present in the test series with powdered Nupik PP compared to the artificial soil series (relative value: 111%). A *t*‐test of the results returned *p* > 0.05, showing that the two samples were not significantly different from each other. Consequently, it can be concluded that no significant negative effect on the reproduction of earthworms was observed when adding 1 g kg^−1^ (dry weight basis) powdered VPP or Nupik PP cups.

## 4. Discussion

The field tests showed that the Nupik cups tested did not undergo significant abiotic degradation when exposed to 14 months of Dorset, UK temperate climate. The CI did not change appreciably (figure 7) but the average molecular weight of the polymer chains did decrease, losing 82% of its value during that time period (table 2). This molecular change was not enough to cause the cup to significantly disintegrate. Even after 24 months, the Nupik cups were mechanically intact. This is in contrast to the accelerated laboraory tests specified by PAS 9017:2020, where the same plastic degraded into microplastics in 28 days. However, in the latter, the CI only reached a value of 0.43. This is below the value of 1.0 required by PAS 9017:2020. Unlike the VPP cups, the Nupik cups’ CI seemed to threshold at that value. It is not clear why this occurs, although the average molecular weight mirrored this behaviour. Again, unlike VPP cups, the Nupik cups reached a lower level of abiotic degradation (table 2).

A clue to explaining this behaviour may lie in the shape of the DSC curves. The endotherm peaks of Nupik control cups and Nupik field trial cup samples are similar, indicating a similar degree of crystallinity, while the artificially aged samples behaved differently in both VPP and Nupik samples, exhibiting a bifurcation of peaks. This may indicate that a shorter chain crystalline phase is forming. It also suggests that a different mechanism of degradation is occurring within the material [13,14]. This could explain, in part, why PAS 9017:2020 is not predictive of real-world abiotic degradation of PAC plastics.

Since the Nupik cups did not significantly degrade in field tests even after 24 months, it is clear they also did not biodegrade. The Nupik cups also failed our laboratory tests of biodegradability, performing no differently than VPP cup. This is in line with previous results from other laboratories, as noted by Sciscione *et al*. [1]. However, there are some studies that claim successful tests of biodegradability of PAC plastics [15–21]. Moreira *et al.* [22]. detected the formation of polyethylene microparticles or polyethylene waxes during UV-accelerated weathering test by using drop point testing and molecular weight analysis [22]. These waxes are assumed to be biodegradable as stated by PAS 9017:2020, although to our knowledge, there have been no studies that confirm this.

Microcracking was observed during the abiotic degradation of Nupik cups in the laboratory tests, leading to the formation of microplastics. It is not yet clear from the literature how problematic microplastics are to biological organisms. Our earthworm avoidance tests and earthworm reproduction tests carried out in artificial soil with powdered Nupik plastic at a concentration of 1 g kg^−1^ showed no significant adverse effects. However, we recommend further investigation, as the Nupik microplastics contain transition metals (table 1), some of which are known to be toxic to bacteria. If such products were to enter soils in high volumes, it is not clear whether there could be an accumulation of transition metals in biological organisms.

Recently, Purkiss *et al*. [23] showed that the majority of home-compostable plastics did not fully biodegrade in real-world tests, even though the manufacturers had carried out certified laboratory testing. The reason for the mismatch between accelerated laboratory tests and field tests is that biodegradable plastics experience different conditions in real-world settings compared to the laboratory, in terms of the key variables that determine their rate of biodegradation, such as temperature, humidity, pH, etc. The abiotic degradation step of the PAS 9017:2020 laboratory test has been correlated with outdoor exposure from the South of France and Florida; however, our results show that the rates of abiotic degradation are lower in the cooler and wetter climate of Dorset, UK. This is evidence that PAS 9017:2020 is not predictive of the biodegradability of PAC plastics in the open unmanaged environment in the UK.

Studies of compostable plastics show that the rate of biodegradation is affected by the format of the product, particularly variables such as thickness and surface-to-volume ratio. We expect this to be true for PAC plastics as well. We tested a PAC plastic cup, but there are other formats in which PAC plastics are sold, such as films, which might abiotically degrade faster. We chose to test a cup in the first instance partly because it is representative of the formats being sold to the public (e.g. at sports grounds). We recommend that further field tests on different formats be carried out to test their behaviour in real-world conditions in the UK. Even within the single-cup format, we noticed differences in colouring between cup batches. These seem to be associated with different concentrations of pro-oxidants (transition metals) (table 1). We speculate that this could be owing to a lack of appropriate quality control by the cup manufacturers, perhaps during the mixing of the master batch into the polymer blends. If this is true, then standards such as PAS 9017:2020 need to include a method to test this variability and ensure it is below defined levels.

Taking all these factors into account we recommend that field tests of PAC plastics in their various product formats be carried out in all territories in which they are being sold. This is the best way for governments to assess the claims of biodegradability. This should be done instead of using PAS 9017:2020, as current evidence, including our own, shows that there is doubt about its ability to predict the real-world behaviour of the PAC plastics.

## 5. Conclusion

- PAS 9017:2020 does not predict the real-world behaviour of the PAC plastics we tested in the open unmanaged environment of the UK temperate climate. Thus, our conclusion is that this British Standard is inadequate as a measure of biodegradability of PAC plastics for the UK climate;
- the PAC plastics we tested do not abiotically degrade or biodegrade in the UK temperate climate;
- the PAC plastics we tested do form microplastics containing transition metals which may be harmful to biological organisms, but at concentrations of 1 g kg^−1^, they show no danger to earthworms; and
- any future version of PAS 9017 should include field testing if it is proved useful as a method to assess the real-world behaviour of PAC plastics.

## Data Availability

All our data is included in the nine figures, four tables, 14 electronic supplementary figures and four electronic supplementary tables [24].
